# Neuroscience Education Begins With Good Science: Communication About Phineas Gage (1823–1860), One of Neurology’s Most-Famous Patients, in Scientific Articles

**DOI:** 10.3389/fnhum.2022.734174

**Published:** 2022-04-28

**Authors:** Stephan Schleim

**Affiliations:** Faculty of Behavioral and Social Sciences, Theory and History of Psychology, Heymans Institute for Psychological Research, University of Groningen, Groningen, Netherlands

**Keywords:** brain damage, ventromedial prefrontal cortex, neurorehabilitation, neuroplasticity, science communication, neuroethics, neuropsychology, phrenology

## Abstract

Phineas Gage is one of the most famous neurological patients. His case is still described in psychology textbooks and in scientific journal articles. A controversy has been going on about the possible consequences of his accident, destroying part of his prefrontal cortex, particularly with respect to behavioral and personality changes. Earlier studies investigated the accuracy of descriptions in psychology textbooks. This is, to my knowledge, the first analysis of journal articles in this respect. These were investigated with regard to four criteria: Description of (1) personality changes, (2) psychopathy-like behavior, (3) alternative explanations besides the immediate brain damage, and (4) Gage’s recovery. 92% of articles described personality changes, 52% of a psychopathy-like kind; only 4% mentioned alternative explanations and 16% described Gage’s recovery. The results are discussed in the light of the available historical evidence. The article closes with several suggestions on improving science communication about the famous case.

## Introduction

**FIGURE 1:**
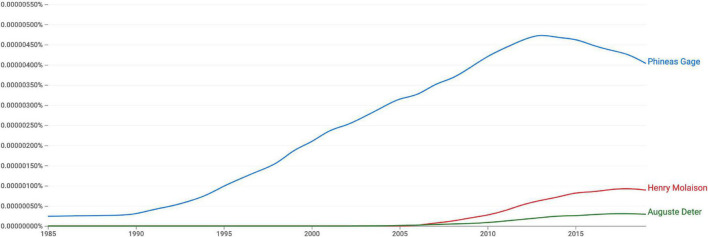
This Google Ngram for English books from 1985 to 2019 shows a steeply increasing interest in Gage’s case since the early 1990s (blue line). Two of the other “essential landmark case reports” discussed by Benjamin et al. (2018), Auguste Deter and Henry Molaison, are shown for comparison (green and red line, respectively). The other clinical cases discussed by these authors (Louis Victor Leborgne and Solomon Shereshevsky) received less attention in English books (not shown on the graph). Source: https://books.google.com/ngrams.

Phineas Gage is one of the most famous patients in the history of neurology, neuropsychology, and clinical neuroscience. On September 13, 1848, the then 25-year-old railroad worker prepared an explosion south of the village of Cavendish, Vermont (United States). When the blast was triggered accidentally, it propelled a heavy iron rod through his skull, irreversibly destroying part of his frontal lobe. Gage’s survival invited investigation and discussion by many medical doctors, brain researchers, and psychologists ever since. And Gage did not only survive: He reportedly stayed conscious and responsive as colleagues brought him home and John M. Harlow, the local physician, started treatment (Harlow, 1848). What keeps fascinating researchers until the twenty-first century are, first, personality changes due to brain damage, and, secondly and more recently, the possibility of Gage’s recovery (Macmillan, 2008; Macmillan and Lena, 2010). Only recently, his case was chosen as the first of six “essential landmark case reports” for neuropsychiatry (Benjamin et al., 2018). An increasing interest since the 1990s can be also seen in books (Figure 1).

The aim of this Research Topic is to improve neuroscience education for the public^1^. Neuroscience education relies on good science communication, that is, that knowledge about the brain as well as its limitation is disseminated in a reliable, comprehensive, and correct way. Earlier reports suggested that the common account that the accident transformed Phineas Gage from a reliable foreman into a psychopath is not based on historical facts, or at least grossly exaggerated (Macmillan, 2000, 2002; Kihlstrom, 2010; Schleim, 2012). Malcom Macmillan, who compiled and reviewed the available historical evidence in detail, summarized such descriptions as follows:

“The composite of modern writers has the accident transforming this Phineas into a restless, moody, unpredictable, untrustworthy, depraved, slovenly, violently quarrelsome, aggressive and boastful dissipated drunken bully, displaying fits of temper, and with impaired sexuality. He is a waster: unwilling to work and unable to settle down. He spends most of the rest of his life in traveling circuses or drifting around fairgrounds to exhibit himself as a human freak, and dies penniless” (Macmillan, 2008, p. 838).

A review of psychology textbooks from the late twentieth and early twenty-first century found many inaccuracies and omissions (Macmillan, 2000; Griggs, 2015). To my knowledge, no one thus far investigated the case’s representation in scientific journal articles. Is this relevant? This issue is not just about scientific accuracy, a value of its own. As we have seen, Gage’s accident still plays a prominent role in medical and psychological education; it is also still featured in public media, after more than 150 years^2^. More importantly, it may inform patients and their relatives about the possible impact of (prefrontal) brain damage and the chances of recovery. The description that Gage was irreversibly turned into a psychopath (or anything near enough) might stigmatize patients and their families and even lead to a self-fulfilling prophecy, if people are then excluded and denied treatment as hopeless cases. Earlier research on science communication has shown that in particular clinical populations are likely to link information about the brain to their personality (O’Connor and Joffe, 2013, 2014; Davis, 2020). In extreme cases, some might even demand to put these allegedly dangerous patients into preventive detention, a possible future scenario described by Raine (2013).

For a more reflective investigation, descriptions about Gage’s accident, its psychological consequences, and his recovery can be discussed with respect to the following three theoretical concepts: (1) Neurodeterminism; (2) localizationism; and (3) neurorehabilitation. The first means that people’s behavior is primarily or solely determined by their brain, not by their situation or environment; the second means that personality traits predisposing people to show certain behaviors can be linked to identifiable areas in the brain^3^; and the third means that people can (at least partially) recover from brain damage, enabled by neuroplasticity and a facilitating environment. To understand science communication about Phineas Gage, I investigated the case’s description in scientific journal articles, as described in the next section. Characteristic quotes are provided in Supplementary Material. I will summarize the findings in the discussion and close with a suggestion on how to communicate better about Gage in the future.

## Investigation

Journal articles covering Phineas Gage were identified on the *Web of Science*, a popular science database featuring more than 80,000,000 records in more than 20,000 journals^4^. A topic search yielded 59 records published from 1994 to 2020 of which 32 were eligible for analysis^5^. These articles were investigated with respect to the described personality changes, particularly whether they referred to psychopathy-like behaviors such as pathological lying, aggressiveness, and violence, and recovery of Gage after the accident; it was also investigated whether they addressed other possible causes of his personality change, such as post-traumatic stress, physical disfigurement, or progressive brain disease.

The result of the analysis is that seven out of the 32 articles are historical overviews which are difficult to assess according to the proposed criteria (Barker, 1995; Neylan, 1999; Macmillan, 2008; Wilgus and Wilgus, 2009; Macmillan and Lena, 2010; Schleim, 2012; Griggs, 2015). These publications quote and compare various historical sources, point out uncertainties, and sometimes even critically appraise that some authors might have seen Gage’s symptoms in the light of the theories they favored. For example, Barker (1995) discusses that Harlow, Gage’s physician, was inclined toward phrenology, an early and extreme form of localizationism, while the renowned Harvard surgeon Henry J. Bigelow, with whom Gage spent some two months, roughly a year after the accident, was a known antilocalizationist. This is important context information when reading that the latter declared Gage completely restored, physically as well as mentally (Bigelow, 1850), while Harlow described the allegedly permanent personality changes that are still frequently quoted in the contemporary literature (Harlow, 1868).

The remaining 25 articles, though, could be assessed according to the proposed criteria (Supplementary Table 1). Almost all of them (23 of 25, or 92%) wrote that Gage’s personality changed after the accident. This is unsurprising, given that this is what makes the case psychologically interesting, that it links brain, mind, and behavior already in a time when modern brain imaging was unavailable. The two exceptions were focusing on the anatomical details (Kelley et al., 2007) or only superficially referred to Phineas Gage, in spite of mentioning his name in the title (Dunbar, 2009). About half of the articles (13 of 25, or 52%) emphasize psychopathy-like behaviors like frequent lying, insulting people, and/or violence. Many did quote from Harlow’s original paper describing personality changes in that direction (Harlow, 1868), but without mentioning other sources or that this evidence is circumstantial.

Two articles explicitly addressed psychopathy in the context of brain damage similar to Gage’s (Thiebaut de Schotten et al., 2015; Reber and Tranel, 2017) and a third one addressed the topic, but concluded “that the supposed psychopathic traits are not evident” in Gage’s case (Kotowicz, 2007: 116). This was also the only paper (1 of 25, or 4%) addressing physical disfigurement and the possibility of social exclusion as an explanation of Gage’s immoral behavior. Finally, a small minority of the articles (4 of 25, or 16%) reported that Phineas Gage found a new job after the accident and had a somewhat stable life. After this brief summary of the results, they will be discussed in more detail the next section.

## Discussion

The aim of this article is to provide an overview of the presentation of Phineas Gage’s accident and its consequences, particularly with respect to his personality, in scientific journal articles. As mentioned in the introduction, earlier publications suggested that his case is not always presented accurately and, in particular, that Gage’s personality changes were sometimes grossly exaggerated (Macmillan, 2000, 2002; Kihlstrom, 2010; Schleim, 2012). An analysis of psychological textbooks found that their descriptions should be improved in several respects (Macmillan, 2000; Griggs, 2015). This article is, to my knowledge, the first overview of scientific journal articles covering Phineas Gage.

The vast majority of the articles described that Gage’s personality changed as a consequence of the accident, irreversibly damaging part of his prefrontal cortex^6^. In my view it *is* likely that he behaved differently afterward. Unfortunately, though, no complex neuropsychological investigation was available in 1848 and Harlow’s detailed account was compiled some 20 years after the event, eight years after Gage’s death, and provides only a very general and in many respects vague account of his personality (Harlow, 1868). This is in stark contrast to Bigelow’s portrayal of Gage as fully recovered (Bigelow, 1850). As mentioned above, both could have been influenced by their belief in localizationism or antilocalizationism (Barker, 1995). Furthermore, Bigelow, the Harvard surgeon, investigated Gage roughly a year after the accident, most of which the patient had spent with his family for recovery after his health state had become stable. By that time, his personality and behavior might have improved, at least partially. This assumption makes more sense when considering alternative effects on Gage’s personality and behavior: We now know that lesser accidents and illnesses than what the young railroad worker went through can have traumatic effects. Actually, many of Gage’s contemporaries imagined him not to survive his injury and even his family is reported to have begged Harlow to let him die (Barker, 1995). And while Kotowicz’s (2007) suggestion that Gage’s physical disfigurement might have led to stigmatization and social exclusion seems exaggerated now that photographs of the recovered patient have been found (Wilgus and Wilgus, 2009; Macmillan and Lena, 2010), the young man *might* have looked like what some of us would call a “zombie,” immediately after the accident, with part of his skull shattered, his left eye permanently damaged, and after Harlow’s surgery (Harlow, 1848). This might, in turn, have influenced how Gage’s friends and former employees reacted to him. The latter reportedly turned down his request to work for the railroad company again, which might have provoked the impulsive behavior and insults Harlow reported (Harlow, 1868).

We will probably never know the whole truth. But the perspective we take will influence the plausibility of neurodeterminism, as described above. What is more based on historical facts, though, is Gage’s recovery. In contrast to some descriptions, he did find new jobs, for example at a farm where he worked with horses. After he moved to Chile, he worked as a stagecoach driver, following a rigorous working scheme, dealing with passengers and caring for the horses (Barker, 1995; Macmillan, 2000, 2002). On the basis of this evidence and more recent knowledge of neurorehabilitation, Macmillan and Lena hypothesize that such highly structured environments, i.e., animals are in need of regular care, traveling schedules have to be followed reliably, facilitated Gage’s recovery (Macmillan, 2008; Macmillan and Lena, 2010). They also found the historical record of a doctor in South America who stated that Gage “was in the enjoyment of good health, with no impairment whatever of his mental faculties” (Macmillan and Lena, 2010, p. 648). It goes without saying that this witness did not know Gage before the accident, just like Bigelow. But we may assume that these medical experts would have noticed signs of pathological lying, aggressiveness, or violence.

This analysis is limited in several respects. First of all, I only investigated journal articles listed on the *Web of Science*. Much of science communication takes place in book chapters in edited volumes and non-fiction books written for broader audiences (Figure 1). Authors might have fewer constraints in such media, such as strict word limits, and thus describe cases in more detail and from more perspectives than is possible in journal articles and psychology textbooks. Secondly, my criteria are pragmatic, about personality changes in general, psychopathy-like changes more particularly, alternative perspectives, and Gage’s recovery. They can be improved to allow a more in-depth analysis of the articles, but still yielded meaningful differences (Supplementary Table 1). With more detailed criteria, it might also be possible to classify the seven “historical overviews” better (see Supplementary Material). Finally, the concepts of psychopathy and psychopathy-like behavior were used in a vague manner here. It should be noted, though, that psychopathy is not a recognized category in the Diagnostics and Statistics Manual of the American Psychiatric Association (2013), although it is used by forensic psychologists and psychiatrists, and that there is an ongoing discussion about its precise definition (e.g., Pickersgill, 2014; Schleim, 2015).

In a similar review, Griggs found that 21 out of 23 introductory psychology textbooks included a discussion of Gage’s case and described it in a generally accurate way, but that only about half of them addressed his subsequent history and recovery (Griggs, 2015). Based on this analysis of scientific journal articles, science communication about Phineas Gage can be improved in several ways: First, different historical sources should be mentioned (particularly Bigelow, 1850; Harlow, 1868), whenever possible, and it should be stated that evidence about Gage’s personality changes is scarce, circumstantial, and controversial. Second, it should be recognized that (at least transitory) psychological trauma and physical disfigurement might have played a role, too, and that Gage suffered from severe infection, fever, and coma shortly after the accident as well as progressive brain damage causing epileptic fits in the long run, which is also the official cause of his premature death in 1860 (Harlow, 1868). Third, Gage’s (at least partial) recovery should be mentioned, to also give patients presently suffering from similar brain damage and their relatives more hope and to stimulate new developments in neurorehabilitation.

## Data Availability Statement

The original contributions presented in the study are included in the article/Supplementary Material, further inquiries can be directed to the corresponding author/s.

## Author Contributions

The author confirms being the sole contributor of this work and has approved it for publication.

## Conflict of Interest

The author declares that the research was conducted in the absence of any commercial or financial relationships that could be construed as a potential conflict of interest.

## Publisher’s Note

All claims expressed in this article are solely those of the authors and do not necessarily represent those of their affiliated organizations, or those of the publisher, the editors and the reviewers. Any product that may be evaluated in this article, or claim that may be made by its manufacturer, is not guaranteed or endorsed by the publisher.

## Footnotes

1
https://www.frontiersin.org/research-topics/16682/how-to-improve-neuroscience-education-for-the-public-and-for-a-multi-professional-audience-in-differ#overview

2 An excellent example is Sam Kean’s article in *Slate*, https://slate.com/technology/2014/05/phineas-gage-neuroscience-case-true-story-of-famous-frontal-lobe-patient-is-better-than-textbook-accounts.html (accessed June 30, 2021). A more recent example is Katie Serena’s article for *all that*’*s interesting* of October 2021 which even described Gage’s case as having helped to “give birth to modern neuroscience,” https://allthatsinteresting.com/phineas-gage (accessed February 9, 2022).

3 Note an important difference between the first two: If localizationism is true, neurodeterminism is also true (at least with respect to personality traits), but not the other way around. Localizationism predicts that people with damage in the same areas have (more or less) the same deficits. But neurodeterminism could be true without it: Then the personality traits or behaviors would depend on the functioning of larger networks in a more holistic fashion, which would make the patients’ expected deficits more diverse and also make it seem more likely that lost functions can be compensated by other parts of the nervous system. It goes without saying that such theoretical thoughts are limited in that in actual clinical cases no two brain lesions will be *exactly* alike.

4 https://clarivate.com/webofsciencegroup/solutions/web-of-science/ (accessed June 30, 2021).

5 The topic search with the phrase “Phineas Gage” covered titles, abstracts, and keywords on the *Web of Science*. Excluded were book reviews, meeting abstracts, letters to the editor, and articles not published in English.

6 There has been a controversy on which brain regions precisely were damaged. Damasio et al. (1994), who made Gage’s case more popular in the recent decades, investigated his skull and concluded that his ventromedial prefrontal cortex must have been destroyed bilaterally. Later reconstructions concluded, though, that only the left part could have been damaged (Ratiu et al., 2004; Van Horn et al., 2012). This discussion is relevant to localizationism, but not for the main topic of this article. It should be noted, though, that Harlow’s surgical treatment and the ensuing infection is likely to have damaged additional brain tissue (Harlow, 1848).
